# Supplementary material for: Evaluation of [18F]MK-6240 binding to tau protein in postmortem human brains of Down syndrome and Alzheimer’s disease and assessment of off-target (non-tau) binding
Source: Acta Neuropathol Commun. 2026 Mar 14;14:100. doi: 10.1186/s40478-026-02267-1 (PMC13107810; doi:10.1186/s40478-026-02267-1)
Supplement: Supplementary file 1 — Supplementary Material 1 [file 40478_2026_2267_MOESM1_ESM.docx]

Evaluation of [^18^F]MK-6240 binding to tau protein in postmortem human brains of Down syndrome and Alzheimer’s disease and assessment of off-target (non-tau) binding

Fariha Karim*^1^, Agnes P. Biju^1^, Christopher Liang^1^, Camryn J. Santos^1^, Maharishi Rajarethenam^1^, Jogeshwar Mukherjee*^1^

[fkarim1@uci.edu](mailto:fkarim1@uci.edu) (F.K.); [apbiju@uci.edu](mailto:apbiju@uci.edu) (A.P.B); [liangc@uci.edu](mailto:liangc@uci.edu) (C.L.); [camrynjs@uci.edu](mailto:camrynjs@uci.edu) (C.J.S); [mrajaret@uci.edu](mailto:mrajaret@uci.edu) (M.R.); [mukherjj@hs.uci.edu](mailto:mukherjj@hs.uci.edu) (J.M.)

^1^Preclinical Imaging, Department of Radiological Sciences,

University of California-Irvine, Irvine, CA 92697, USA.

**Radiosynthesis of [^18^F]MK-6240**

All solvents were purchased from Sigma-Aldrich, St. Louis, MO, USA. Radiosynthesis of [^18^F]MK-6240 was carried out using modifications of reported procedures [1]. High specific activity fluorine-18 radioactivity was purchased from PETNET, Inc (Culver City, CA, USA) and counted in a Capintec CRC-15R dose calibrator (Florham Park, NJ, USA). This hydrogen [^18^F]fluoride in H_2_^18^O was passed through light QMA Sep-Pak (Waters Corp., Milford. MA, USA), preconditioned with 2 mL of potassium carbonate (Sigma-Aldrich, St. Louis, MO, USA), 140 mg/mL, followed by 2 mL of anhydrous acetonitrile. The trapped [^18^F]fluoride in QMA was eluted with 2.5 mL of Kryptofix 2.2.2 (Sigma-Aldrich, St. Louis, MO, USA)/potassium carbonate solution (36 mg Kryptofix and 7.5 mg potassium carbonate in 2.4 mL acetonitrile and 0.1 mL water) and transferred to the reaction vessel. The initial drying step of the [^18^F]fluoride, Kryptofix 2.2.2., and K_2_CO_3_ mixture was at 120^o^C for 10 min under a stream of nitrogen gas. The [^18^F]fluoride solution was further dried with acetonitrile (2x1 mL) at 120 ^o^C for 7 min. To the dried [^18^F]fluoride reaction mixture, [^18^F]MK-6240 precursor, *N*-[(tert-butoxy)carbonyl-*N*-(6-nitro-3-[1H-pyrrolo[2,3-c]pyridine-1-yl)isoquinolin-5-yl)carbamate (1ClickChemistry, Inc., New Jersy, USA) 2 mg dissolved in 1 mL dimethylformamide (DMF) was added. This reaction mixture was heated at 160 ^o^C for 20 min and then cooled. Methanol (5 mL) was added to the reaction vial and the contents were passed through neutral alumina Sep-Paks (Waters, Inc., Milford. MA, USA), prewashed with methanol. The DMF-methanolic containing [^18^F]MK-6240 was evaporated *in vacuo* followed by semipreparative HPLC (Gilson, Inc., Middleton, WI, USA) purification using an Alltech C_18_ reverse-phase column (10 μm, 250x10 mm) and UV detector (254 nm), mobile phase: 60% acetonitrile-40% 0.1% aqueous triethylamine, 2.5 mL/min, r.t. = 22 min. [^18^F]MK-6240 was made in modest yields (30% decay corrected) in specific activities of 74 GBq/μmol. The collected fraction was taken to near dryness *in vacuo* and final product was taken up in 10% alcohol in sterile saline.

Radiosynthesis of [^125^I]IPPI was carried out as previously reported [2].

**References**

[1] Collier TL, Yokell DL, Livni E, et al. (2017) cGMP production of the radiopharmaceutical [ 18  F]MK 6240 for PET imaging of human neurofibrillary tangles. J Labelled Comp Radiopharm 60(5):263-269.

<https://doi.org/10.1002/jlcr.3496>

[2] Mukherjee J, Liang C, Patel KK, Lam PQ, Mondal R (2021) Development and evaluation of [125I] IPPI for Tau imaging in postmortem human Alzheimer&#39;s disease brain. Synapse 75(1):e22183. <https://doi.org/10.1002/syn.22183>
